# Papillary Muscle Involvement during Acute Myocardial Infarction: Detection by Cardiovascular Magnetic Resonance Using T1 Mapping Technique and Papillary Longitudinal Strain

**DOI:** 10.3390/jcm12041497

**Published:** 2023-02-14

**Authors:** Giacomo Pambianchi, Martina Giannetti, Livia Marchitelli, Giulia Cundari, Viviana Maestrini, Massimo Mancone, Marco Francone, Carlo Catalano, Nicola Galea

**Affiliations:** 1 Department of Radiological, Oncological and Pathological Sciences, Sapienza University of Rome, 00161 Rome, Italy; 2 Department of Clinical, Internal, Anesthesiological and Cardiovascular Sciences, Sapienza University of Rome, “Policlinico Umberto I” Hospital, 00161 Rome, Italy; 3 Department of Biomedical Sciences, Humanitas University, Via Rita Levi Montalcini 4, Pieve Emanuele, 20072 Milan, Italy; 4 IRCCS Humanitas Research Hospital, Via Manzoni 56, Rozzano, 20089 Milan, Italy

**Keywords:** myocardial infarction, papillary muscles, cardiac magnetic resonance, T1 mapping, myocardial strain

## Abstract

Papillary muscle (PPM) involvement in myocardial infarction (MI) increases the risk of secondary mitral valve regurgitation or PPM rupture and may be diagnosed using late gadolinium enhancement (LGE) imaging. The native T1-mapping (nT1) technique and PPM longitudinal strain (PPM-ls) have been used to identify PPM infarction (iPPM) without the use of the contrast agent. This study aimed to assess the diagnostic performance of nT1 and PPM-ls in the identification of iPPM. Forty-six patients, who performed CMR within 14–30 days after MI, were retrospectively enrolled: sixteen showed signs of iPPM on LGE images. nT1 values were measured within the infarcted area (IA), remote myocardium (RM), blood pool (BP), and anterolateral and posteromedial PPMs and compared using ANOVA. PPM-ls values have been assessed on cineMR images as the percentage of shortening between end-diastolic and end-systolic phases. Higher nT1 values and lower PPM-ls were found in infarcted compared to non-infarcted PPMs (nT1: 1219.3 ± 102.5 ms vs. 1052.2 ± 80.5 ms and 17.6 ± 6.3% vs. 21.6 ± 4.3%; *p*-value < 0.001 for both), with no significant differences between the nT1 of infarcted PPMs and IA and between the non-infarcted PPMs and RM. ROC analysis demonstrated an excellent discriminatory power for nT1 in detecting the iPPM (AUC = 0.874; 95% CI: 0.784–0.963; *p* < 0.001). nT1 and PPM-ls are valid tools in assessing iPPM with the advantage of avoiding contrast media administration.

## 1. Introduction

The left ventricular papillary muscles (PPMs) are intracavitary muscular structures arising from the ventricular wall, modulating the function of the mitral valve (MV) [1].

The PPM involvement during myocardial infarction (MI) represents one of the most frequent causes of ischemic secondary MV regurgitation (MVR, 30–50% of the cases) and independently increases the mortality rate [2].

PPMs have peculiar vascular anatomy: posteromedial (PM) PPM has a single arterial supply (63% of the population), commonly by the right coronary artery (RCA) posterior descending branch in the case of a right-dominant system, or by the left circumflex artery (LCX) or obtuse marginal branch (OM) in a left-dominant system. Conversely, the blood supply of the anterolateral (AL) PPMs group is generally dual (71% of the population), both from LCX/OM and the left anterior descending artery (LAD) through the first diagonal branch [3]. This results in a more frequent ischemic involvement of the PM-PPM compared to the AL-PPM (77% vs. 26%) [2], with high incidence (26–53%) and prevalence (40–45%) of papillary muscle infarction (iPPM) after acute MI [4,5].

The ischemic injury of PPMs causes impairment of its contractile function and a reduction in systolic shortening (PPMs dysfunction) and may be related to MVR, especially when the ventricular function is compromised, resulting in poorer prognosis [6,7].

Eitel I. et al. demonstrated that the presence of iPPM was associated with larger infarcts, less myocardial salvage, impaired left ventricular function, and more pronounced reperfusion injury, with consequent greater risk of major adverse cardiac events [4]. Moreover, iPPM is related to older age, a greater rate of microvascular obstruction (MVO) [8], and left ventricular (LV) function impairment [5].

The detection of iPPM may improve risk stratification and therapeutic decision-making in MI patients.

Transthoracic echocardiography (TTE) may evaluate the kinetics and morphology, of PPMs, which is particularly useful in ruling out PPM rupture. However, the angle dependency of the TTE technique and its intrinsic limitation in tissue characterization hamper the accuracy of detecting iPPM [9].

Cardiac magnetic resonance (CMR) has been established as the best non-invasive modality for iPPM assessment because of the high spatial resolution and intrinsic contrast of soft tissues, enhanced by the use of a gadolinium-based contrast agent (GBCA). The identification of myocardial edema in T2 weighted sequences and necrosis in the late gadolinium-enhanced (LGE) images enables characterization and quantification of the myocardial damage.

Native T1 mapping (nT1) imaging, based on the relaxometric properties of a normal and pathological myocardium, represents a novel quantitative biomarker that allows for the assessment of the severity and extent of injured myocardium, even without GBCA administration [10,11].

It is known that the PPM signal follows the modification of the adjacent myocardium in CMR sequences and nT1 mapping images, however, it has not been established yet if the PPM nT1 mapping values change in accordance with the ventricular wall myocardium during MI and may detect iPPM [3].

As a further advantage of CMR imaging, the assessment of PPM dysfunction by cine-MR images may be implemented by the recent introduction of “tissue tracking” based strain analysis.

The analysis of papillary longitudinal strain (PPM-ls) improves the evaluation of PPM dysfunction, as already demonstrated in many echocardiographic studies, and it could be helpful when iPPM is suspected [12,13,14].

To the best of our knowledge, no study has investigated the diagnostic performance of nT1 mapping and strain analysis in the assessment of PPM involvement in acute MI.

The purpose of our study was therefore to test the diagnostic capability of nT1 mapping sequences and strain evaluation to detect iPPM in a cohort of ST-elevation myocardial infarction (STEMI) patients.

## 2. Materials and Methods

### 2.1. Study Subjects

We retrospectively selected 46 patients who underwent CMR examination between 14 to 30 days after STEMI from 2018 to 2021 and had signs of recent MI at both coronary angiography and CMR (presence of myocardial edema and LGE in the coronary distribution of culprit stenosis). The MI-to-CMR time range was set between 14 and 30 days in order to reduce the edema interference in the myocardial nT1 relaxation time, as it is well-known that edema has a bimodal increase pattern up to 7–10 days after MI, with a progressive decrease in the following days/months [15,16].

The exclusion criteria were:

- Poor CMR image quality (non-diagnostic images for the presence of artifacts or excessive noise) or papillary muscle thickness < 4 mm;
- Known or evidence of prior myocardial infarction or non-ischemic cardiomyopathies at CMR;
- Rapid ventricular response-atrial fibrillation (RVR-AF) or tachyarrhythmias;
- Implantable cardioverter-defibrillator/pacemaker bearers;

Ethical Committee approval and written informed consent from all participants were obtained.

### 2.2. CMR Protocol

All CMRs were performed on a 1.5T scanner (Magnetom Avanto, Siemens Medical System, Erlangen, Germany) using body and phased array coils. The CMR protocol included:

- T2-weighted Short Tau Inversion Recovery (T2-STIR) sequence acquired on the short axis (from base to apex, 8 slices at least), on 2-chamber and 4-chamber views (TR: 2 R-R intervals; TE: 75 ms; FA: 180°; TI: 170 ms; Slice thickness: 8 mm; FoV: 320–400 mm; Matrix: 256 × 256, Voxel size: 2.3 × 1.3 × 8 mm);
- Modified Look-Locker Inversion Recovery sequence for nT1 mapping, acquired on three short-axis slices at basal, mid-ventricular, and apical views and one four-chamber view before the administration of a single dose of 0.15 mMol/Kg gadoterate dimeglumine (TR: 314 ms; TE: 1.12 ms; FA: 12°; Slice thickness: 8 mm; FoV: 340–400 mm; matrix 256 × 256; Voxel size: 1.6 × 1.3 × 8 mm);
- Cine-Steady State Free Precession sequences for cineMR imaging, acquired on the short axis (from the base to the cardiac apex, 10–12 slices) and 2-, 3- and 4-chamber views (TR: 51.3 ms; TE: 1.21 ms; FA: 45°; Slice thickness: 8 mm; Matrix: 256 × 256; FoV: 340–400 mm; Voxel size: 1.6 × 1.3 × 8.0 mm);
- Contrast-enhanced Inversion Recovery T1-weighted images acquired from 15 to 20 min after GBCA injection, during breath-hold at end-diastole in the short axis (from the base to the cardiac apex, 10–12 slices) and then on the 2-, 3- and 4-chamber views (TI: 350–400 ms; TR: 9.6 ms; TE: 4.4 ms; matrix 256 × 208, FA: 25°; Slice thickness: 8 mm; interslice gap: 2 mm) for LGE imaging.

Field inhomogeneity correction, noise reduction, and motion correction were performed by using the algorithms incorporated in the package with standard settings.

### 2.3. Image Analysis

CMR images were analyzed by two experienced radiologists with six and fifteen years of experience using dedicated software (Circle CVI42, Circle Cardiovascular Imaging Inc., Calgary, AB, Canada) in consensus.

First, the overall diagnostic quality of the CMR exam was evaluated to assess the eligibility of each patient. Second, the image quality of the nT1 maps was assessed using a 5-point Likert scale (5, excellent; 4, good; 3, adequate; 2, sufficient; 1, poor/not acceptable) with the focus on image noise, edge conspicuity, motion, and co-registration artifacts, field inhomogeneities, and tuning frequency-offsets.

Ventricular volumes have been measured using cineMR images. In particular, epi- and endocardial LV borders were traced on the short-axis images in a semiautomatic fashion, and body surface area (BSA) was used to index the parameters.

**Figure 1:**
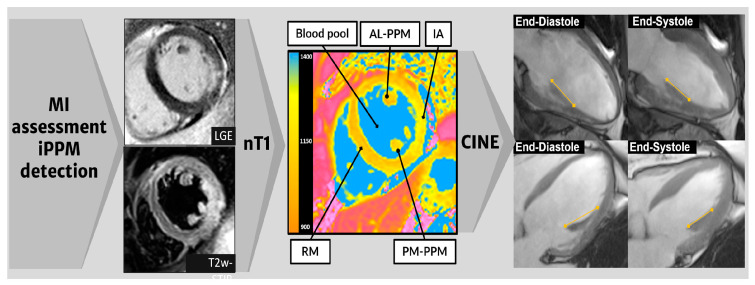
**Scheme of CMR image analysis process.** LGE and T2-STIR images were analyzed to identify IA, RM, BP, and the two PPMs. Then, nT1 was measured by placing multiple ROIs in the previously defined areas. Finally, the cineMR images acquired on long axis views (two chambers, in the upper row, and four chambers below) in the end-diastolic and end-systolic phases were evaluated to calculate the PPM-ls. *CMR: cardiac magnetic resonance; LGE: late gadolinium enhancement; STIR: short tau inversion recovery; BP: blood pool; RM: remote myocardium; IA; infarcted area; AL: anterolateral; PM: posteromedial; PPM-ls: PPM longitudinal strain; ROI: region of interest*.

Short- and long-axis T2-STIR and LGE images were used to individuate the infarcted area (IA), area at risk (AAR), non-ischemic remote myocardium (RM), and intraventricular blood pool (BP) as previously described [17] (Figure 1). Using T2-STIR and LGE as a guide, multiple regions of interest (ROIs) were traced on nT1 maps to calculate the nT1 values of IA, RM, and BP. Further ROIs were placed within the AM- and PM-PPMs to measure the nT1 values, with an offset of 10% of the outer borders, taking the average value out of three measurements. When MVO was evident in the LGE images, it was excluded from the ROI for the IA nT1 measurement. An additional ROI was placed within the thoracic muscle that was best represented in the image (i.e., latissimus dorsi or pectoralis), to measure the background noise.

The presence of MVR was detected in echocardiographic exams and during the CMR exam and classified as no-regurgitation, mild, moderate, and severe.

All patients were classified into two groups (iPPM and niPPM) based on the presence or absence of iPPM, respectively.

iPPM was defined by the presence of all of the following three criteria:

1. Hyperintense area within AM- or PM- PPM on T2-STIR images (short and/or long axis views);
2. Hyperintense area within AM- or PM- PPM on LGE images (short and/or long axis views);
3. Obstructive coronary artery disease at coronary angiography in the culprit vessel (RCA-posterior descending branch or LCX for the PM-PPM, LCX, or ADA for the AM-PPM).

The systolic and diastolic length of the PPMs were assessed on cineMR images acquired on the horizontal and vertical long-axis views, and then the PPM longitudinal strain (PPM-ls) was calculated using the Lagrange strain formula, as follows [18]:

$$\mathrm{PPM}\text{-}\mathrm{ls}=\frac{\left(\mathrm{end}\;\mathrm{diastolic}\;\mathrm{PPM}\;\mathrm{length}\;-\;\mathrm{end}\;\mathrm{systolic}\;\mathrm{PPM}\;\mathrm{length}\right)}{\mathrm{end}-\mathrm{diastolic}\;\mathrm{PPM}\;\mathrm{length}}\%$$

The contrast-to-noise ratio (CNR) of the nT1 values between IA and BP/RM and between iPPM and BP/RM was also quantified according to the following formulas:

CNR(IA/BP) = |(nT1_(IA)_ − nT1_(BP)_)|/SD_(Noise)_

CNR(IA/RM) = |(nT1_(IA)_ − nT1_(RM)_)|/SD_(Noise)_

CNR(iPPM/BP) = |(nT1_(iPPM)_ − nT1_(BP)_)|/SD_(Noise)_

CNR(iPPM/RM) = |(nT1_(iPPM)_ − nT1_(RM)_)|/SD_(Noise)_

where SD_Noise_ is the standard deviation measured in a thoracic muscle ROI. Then, we compared the CNR(IA/BP) vs. CNR(iPPM/BP) and CNR(IA/RM) vs. CNR(iPPM/RM).

To evaluate the inter-observer variability, the two observers independently measured the nT1 values of the PPMs from 10 STEMI patients with iPPM and 10 STEMI patients without iPPM. One of the two observers measured the nT1 value of the PPMs twice to assess the intra-observer variability. The time delay between two reads for the intra-observer variability was at least 2 weeks.

### 2.4. Statistical Analysis

All of the data were analyzed using SPSS (version 26.0, SPSS, Inc., Chicago, IL, USA). Continuous parameters are presented as mean  ±  standard deviation (SD) and categorical ones are expressed as numbers (%).

To assess the difference between the groups (iPPM vs. niPPM), one-way analysis of variance (ANOVA) and the Student’s T-test were used for continuous variables. The Chi-square test was used to assess the difference for categorical variables. A univariate analysis was performed for the parameters that showed statically significant values. Variables were considered significant at *p* values < 0.05.

The Bland–Altman analysis was used to assess the interobserver reproducibility of the method, and then the intraclass correlation coefficient (ICC) was used to evaluate the intra- and inter-observer variability for measuring the nT1 of the papillary muscle.

Pearson’s correlation coefficient was calculated to evaluate the relationship between the PPMs’ nT1 and the PPMs’ morpho-dynamic measurements (end-systolic/end-diastolic lengths and longitudinal strain).

Variables with a *p*-value < 0.05 in the univariable analysis were included in the multivariable linear regression analysis (stepwise method). A *p*-value < 0.05 was considered statistically significant.

A receiver operating characteristic (ROC) curve was performed to assess the sensitivity and specificity of the nT1 and PPM-ls values in detecting iPPM.

## 3. Results

### 3.1. Patient Characteristics

The final population consisted of 46 patients (38 men) with a mean age of 59.5 ± 12.6, which were classified into the iPPM group (n = 16) and niPPM group (n = 30).

**Figure 2:**
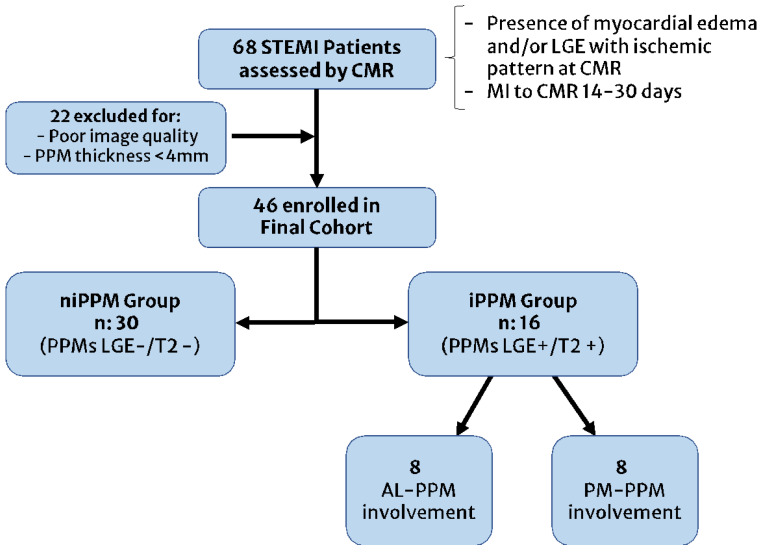
Patients recruitment flowchart. STEMI: ST-elevation myocardial infarction; CMR: cardiac magnetic resonance; LGE: late gadolinium enhancement; MI: myocardial infarction, PPM: papillary muscle; AL: anterolateral; PM: posteromedial.

The patient selection scheme is reported in Figure 2. The patients’ demographics and clinical characteristics are summarized in Table 1.

There was no significant difference in the gender, age, MI-to-CMR interval, body mass index (BMI), presence of systemic arterial hypertension (HTN), diabetes mellitus (DM), smoking, and family history of MI between the two groups.

Most of the patients were male (82.6%), with comparable sex prevalence (88% vs. 80%), age (60 ± 9 vs. 59 ± 14), and onset (17 ± 8 vs. 18 ± 6) between the two groups (*p* > 0.693 for all the variables).

### 3.2. CMR Features

The iPPM and niPPM groups did not show significant differences in terms of LV- and right ventricular (RV) ejection fraction (EF), indexed end-diastolic volume (EDV), end-systolic volume (ESV), and stroke volume (SV), as reported in Table 2.

The nT1 values of RM, IA, and BP did not show significant differences between the two groups (*p*: 0.093–0.517 for all the parameters).

Most of the patients (80%) were not affected by MVR, with no significant difference between the iPPM and niPPM groups (81% vs. 80%; *p*: 0.879). Only eight patients showed mild MVR and one patient from the niPPM group suffered from moderate MVR.

The mean score for the image quality was 4.47 ± 0.62. In particular, the image quality was acceptable in three patients (6.5%), good in 18 (39%), and excellent in 25 (54%) patients.

Infarcted PPMs showed increased nT1 values when compared to the non-infarcted ones (*p* < 0.001), and also when the AL-PPMs and PM-PPMs were separately analyzed (*p* < 0.001 for both).

**Figure 3:**
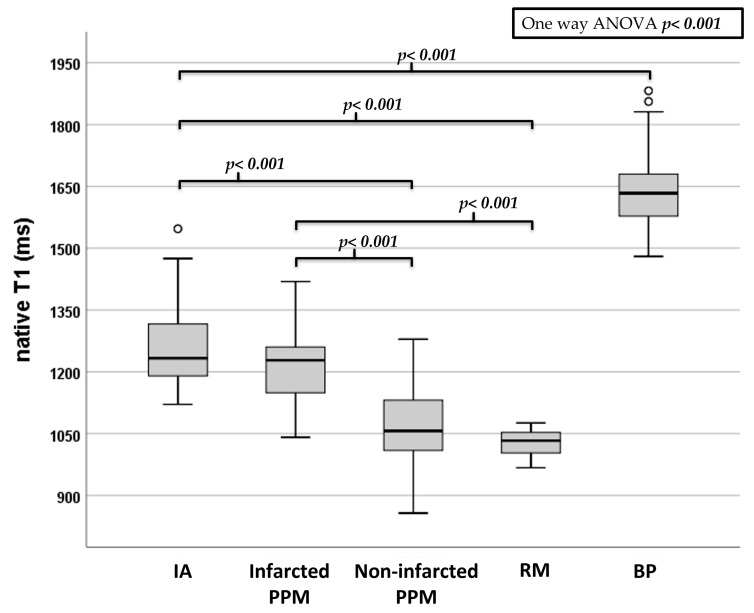
Box plot graph showing the nT1 values of the different ROIs placed in the myocardium and PPMs. Error bars represent the 95% confidence interval (CI), and the top and the bottom of the boxes represent the third and the first quartiles, respectively. *p*-values were calculated with one-way ANOVA and Bonferroni post-hoc analysis and considered significant when <0.05. *ANOVA: analysis of variance; ms: milliseconds; IA: infarcted area; RM: remote myocardium; BP: blood pool*.

The nT1 values of the infarcted PPMs were also higher than those of RM (*p* < 0.001), whereas no significant differences were found between the infarcted PPMs and IA (*p* = 0.657) and non-infarcted PPM and RM (*p*: 0.240), as shown in Figure 3.

The PPMs’ systolic and diastolic length values were comparable between the iPPM and niPPM groups (*p*: 0.457–0.622) as summarized in Table 3.

**Figure 4:**
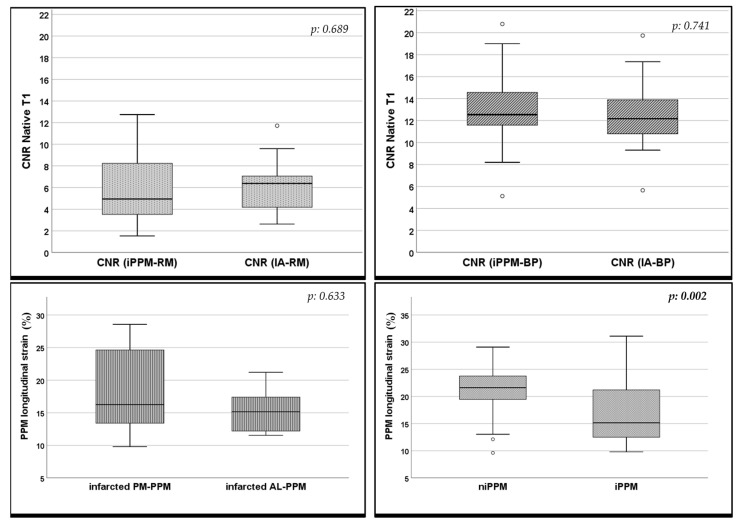
Box plot graphs. The upper row shows the CNR value comparison between CNR (infarcted PPM/RM) and CNR(IA/RM) on the left, and CNR(IA/BP) vs. CNR (infarcted PPM/BP) on the right. The row below shows the PPM-ls comparison between the infarcted AL- vs. PM-PPM and between the infarcted vs. non-infarcted PPMs. Error bars represent the 95% CI, and the top and the bottom of the boxes represent the third and the first quartiles, respectively. *p*-values are calculated with the Student-T test and considered to be significant when <0.05. *CNR: contrast-to-noise ratio; PPM: papillary muscle; IA: infarcted area; RM: remote myocardium; BP: blood pool; AL-PPM: anterolateral papillary muscle; PM-PPM: posteromedial papillary muscle*.

However, infarcted PPMs showed a reduction in PPM-ls when compared to the non-infarcted-PPMs (*p* = 0.002), which was also confirmed in the AL-/PM-PPM subgroup analysis (*p* = 0.009–0.011), as shown in Figure 4.

All of the CNR values calculated in the T1 maps are reported in Table 4. The comparisons between the CNR of the infarcted-PPM and the IA did not show any significant difference for both the/BP and/RM CNRs (*p*: 0.741 and 0.689, respectively), as shown in Figure 4.

**Figure 5:**
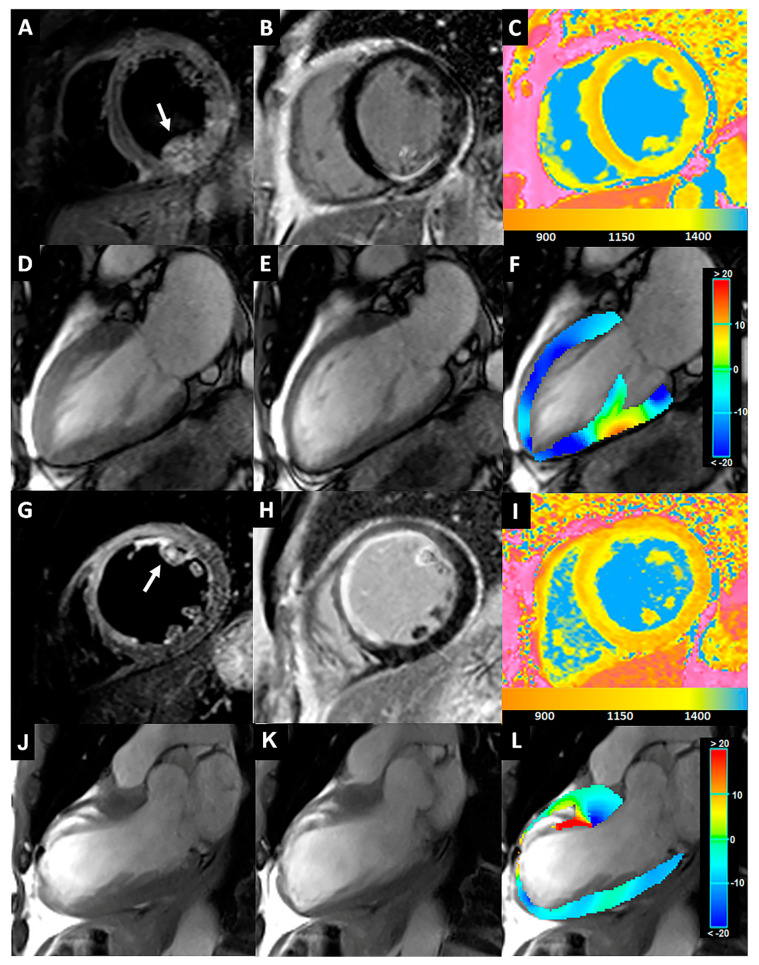
(**A**–**F**) A 55-year-old man with inferior STEMI for RCA occlusion. T2-STIR (**A**) and LGE (**B**) images acquired on the short-axis views revealed LV inferior wall edema and enhancement, respectively, with PM-PPM involvement (**arrow**). In the corresponding nT1 map (**C**), PM-PPM showed a similar increase in the nT1 values compared to the infarcted myocardium (yellow color). The CineMR images acquired on the long-axis view in the end-diastolic (**D**) and end-systolic (**E**) phases showed hypokinesia of the lower wall and impaired contraction (lower shortening and thickening) of PM-PPM, as shown in the colorimetric map of longitudinal strain analysis by the tissue tracking automatic module (**F**). (**G**–**L**) A 61-year-old with extensive anteroseptal STEMI due to LAD proximal occlusion. A large area of transmural myocardial edema and mid-wall/subendocardial enhancement of the anteroseptal LV wall are visible in the T2-STIR (**G**) and LGE images (**H**), respectively, with AL-PPM involvement (arrow). The nT1 map (**C**) demonstrated a strong increase in the infarcted area, included in the AL-PPM (yellow color). AL-PPM is akinetic on the cineMR images acquired on the long-axis view in the end-diastolic (**J**) and end-systolic (**K**) phases, as highlighted by a red color depicting the severely reduced PPM-ls (**L**). *STEMI: ST-elevation myocardial infarction; RCA: right coronary artery; LGE: late gadolinium enhancement; LV: left ventricular; PM: posteromedial; PPM: papillary muscle; LAD: left anterior descending artery; AL: anterolateral; PPM-ls: PPM-longitudinal strain*.

Two cases of iPPM involving the AL- and PM-PPMs are reported in Figure 5.

**Figure 6:**
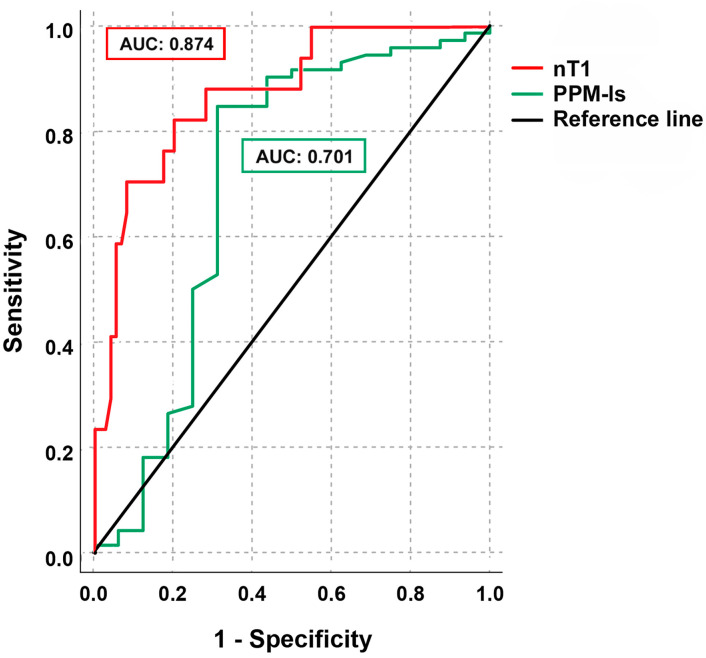
ROC analysis illustrates the diagnostic performance of nT1 and PPM-ls to detect iPPM. AUC: area under the curve, nT1: native T1; PPM-ls: PPM-longitudinal strain.

ROC analysis (Figure 6) showed that nT1 proved to have an excellent discriminatory power with an area under the curve (AUC) of 0.874 (95% confidence interval: 0.784–0.963, *p* < 0.001), and an accuracy rate of 74%. The PPM-ls had an inferior diagnostic performance with an AUC = 0.701 (95% confidence interval: 0.523–0.878). The Youden test was performed to individuate the best nT1 and PPM-ls cut-off values, in order to diagnose the iPPM, which resulted in being 1191.5 (sensitivity: 71%, specificity: 92%) and 17.8% (sensitivity: 85%, specificity: 69%), respectively.

## 4. Discussion

In our study, we investigated the performance of the nT1 mapping sequences and strain evaluation to detect iPPM in the STEMI patients.

The main findings of our study include (1) the good-to-excellent image quality of nT1 maps, with high CNR between iPPMs and RM or intracavitary BP; and (2) the good performance of nT1 and PPM-ls, assessed as longitudinal percent shortening of PPM, in iPPM identification.

Previous reports have demonstrated a high accuracy of nT1 mapping in the diagnosis of MI by using LGE as the reference standard, but there is no evidence of its performance in detecting iPPM as yet [17].

The identification of PPM abnormalities by CMR may be hindered by the inherent limitations of the T2-STIR and LGE sequences, which are prone to motion artifacts and partial volume effects in uncooperative patients and subjects with high or irregular heart rate. Furthermore, the contrast resolution between PPMs and adjacent LV cavity is variably influenced by slow flow phenomena in T2-STIR or high blood signal from persistent gadolinium enhancement in the LGE images [17].

The nT1 mapping technique has been emerging as a valid alternative in the assessment of MI, thanks to its ability to distinguish the intrinsic relaxometric characteristics of the ischemic tissues in comparison with the healthy myocardium, even without the need for GBCA administration. In addition, in the nT1 maps, the motion artifacts were reduced by the application of the motion correction algorithm before curve-fitting and map generation [19,20,21].

In our study, the image quality of nT1 maps was generally good-to-excellent, and the depiction of PPM from the cavity was clear in almost all patients, rarely hampered by artifacts (e.g., B0-field inhomogeneity, and edge artifacts).

Moreover, when the diagnostic quality of the nT1 maps was not optimal, the nT1 relaxometric values of the PPMs were calculated by measuring the signal values of PPM for each inversion time, then generating the T1 signal decay curve.

Quantitative analysis, obtained by calculating the CNR values, revealed similar contrast resolution in differentiating IA and infarcted PPMs from RM or intracavitary BP.

This could be an advantage over the LGE images, where the increasing use of faster protocol or lower GBCA dose may lead to insufficient wash-out of circulating gadolinium or enhancement of infarcted tissue, resulting in suboptimal contrast between the BP and PPMs and the IA size underestimation [17,22,23,24].

Furthermore, we demonstrated an excellent discriminatory power and accuracy rate of nT1 in detecting iPPM, with good sensitivity and high specificity (71%; 92%) for 1191.5 ms as the threshold. On this ground, the measurement of PPM nT1 can be useful when the T2-STIR and LGE images are not clear or affected by artifacts.

Another innovative result provided by our study is the efficiency of PPM-ls analysis in the evaluation of post-ischemic PPMs contractile dysfunction.

Although no differences in PPM length were found in the comparison between infarcted and non-infarcted PPMs, in both the end-systolic and end-diastolic phases, longitudinal percent shortening measured by PPM-ls was significantly different.

This finding suggests that ischemic injury predominantly affects the dynamic properties of PPMs, rather than anatomical ones, as previously demonstrated in echocardiographic studies [25]. Papillary dysfunction, indeed, seems to be determined not only by the necrosis and fibrosis of PPMs, caused by direct ischemic damage, but also by the redistribution of myocardial wall tension forces due to concomitant ventricular wall motion abnormalities [26]. TTE, implemented by speckle tracking, may provide an objective and quantitative assessment of PPMs and MV apparatus with high reproducibility, as demonstrated in patients with mild to moderate rheumatic mitral stenosis. We can assume that the fractional shortening measurement by CMR could represent a valid option, which may be embodied in the standard evaluation of patients with MI [25,27].

The use of T1 mapping sequences and strain analysis has improved the quantitative evaluation of tissue and functional features in the assessment of MI patients, introducing new biomarkers, whose clinical values are yet to be established.

Native T1 mapping could enable the implementation of faster protocols by replacing conventional T2-STIR and LGE sequences without the use of GBCA administration, which could be particularly useful in uncompliant patients or with contraindications to GBCA.

As previously discussed, iPPM may worsen the prognosis for patients with MI because of the risk of acute mechanical consequences such as papillary muscle rupture or chronic PPM dysfunction with the development of post-ischemic MVR [4].

Notably, no significant differences were observed in MVR prevalence between patients with or without iPPM. Although the small sample size of our population limits the generalization of the results, this finding supports the evidence that the pathophysiological link between iPPM and post-ischemic MVR occurrence still needs to be clarified, as various anatomical and functional conditions may be involved [28]. 

Moreover, it should be noted that our patients were examined during the subacute post-infarction phase, and, therefore, a later onset in the chronic phase of MV regurgitation cannot be excluded.

Conversely, in chronic ischemic cardiopathy, the scarring of PPMs and the outward displacement of the PPMs associated with LV wall remodeling may cause the “tethering” of mitral leaflets and result in mitral valve complex deformity and secondary MVR [29,30,31].

The detection of the ischemic involvement of PPMs and subpapillary regions of the LV wall could help to identify patients who are at risk of developing MVR [26,30].

Furthermore, the implication of iPPM in the post-ischemic LV remodeling process is still controversial [2,32,33]. Many studies have demonstrated that multiple factors play a role in this complex adaptative phenomenon by using CMR [34]. However, it is still unknown if iPPM may facilitate adverse remodeling or the redistribution of intracavitary hemodynamic forces, which appears to be one of the leading mechanisms of remodeling [35]. Further studies with follow-up imaging and a larger cohort are needed to clarify these relationships and to assess the real prognostic value of iPPM.

The main limitation of our research was the small sample size of the cohort, especially of the iPPM group, who were analyzed in a single-center retrospective study.

Additionally, we did not perform a quantitative assessment of the ischemic burden in order to explore the relationship between IA extent and iPPM occurrence. In our cohort, all iPPM patients presented an increased signal of infarcted PPM in both T2-STIR and LGE, therefore, the nT1 signal in the isolated edema of PPMs without necrosis (no enhancement in LGE images) was not investigated. Finally, the lack of clinical or CMR follow-up data did not allow us to assess the clinical relevance of this diagnosis, which would require targeted investigations of a larger population with longer follow-up.

## 5. Conclusions

iPPM diagnosis is an ancillary CMR feature, which improves the prognostic stratification in subacute MI patients. nT1 mapping and PPM-ls may be considered as valid tools in detecting iPPM with the advantage of avoiding GBCA administration and is a quantitative approach.

## Figures and Tables

**Table 1:** The patients’ characteristics and clinical data.

Parameter	Total	iPPM	niPPM	*p*-Value
Population (n, %)	46 (100)	16 (35)	30 (65)	-
Age (Years, mean ± SD)	59 ± 13	60 ± 9	59 ± 14	0.815
Sex (n men, %)	38/46 (83)	14/16 (88)	24/30 (80)	0.694
MI-to-CMR interval (days, mean ± SD)	18 ± 7	17 ± 8	18 ± 6	0.917
BMI (kg/m^2^, mean ± SD)	26.1 ± 4.2	25.2 ± 4.6	26.6 ± 3.9	0.581
HTN (n°. %)	30 (65)	10 (63)	20 (67)	0.697
HR (bpm, mean ± SD)	71 ± 5	74 ± 8	70 ± 3	0.031
DM (n°. %)	6 (13)	2 (13)	4 (13)	0.936
Current Smoker (n°. %)	29 (63)	10 (63)	19 (63)	0.943
Family history of MI (n°. %)	15 (33)	6 (38)	9 (30)	0.734

BMI: body mass index; iPPM: infarction of papillary muscle; niPPM: non-infarction of papillary muscles; MI-to-CMR interval: the interval between onset of symptoms and cardiac magnetic resonance; BMI: body mass index; HTN: systemic arterial hypertension; HR: heart rate; DM: diabetes mellitus.

**Table 2:** The CMR features.

Parameter	Total (N: 46)	iPPM Group (N:16)	niPPM Group (N:30)	*p*-Value
MI location anterior/lateral/inferior, n (%)	28/5/13 (61/11/28)	9/1/6 (56/6/38)	19/4/7 (64/13/23)	-
AL-/PL-PPM infarction, n (%)	8 (17)/8 (17)	8 (50)/8 (50)	-	-
LV EDV/BSA (mL/mq)	89.59 ± 26.53	96.01 ± 27.51	86.16 ± 25.79	0.235
LV ESV/BSA (mL/mq)	54.37 ± 23.72	61.41 ± 23.70	50.61 ± 23.24	0.143
LV SV/BSA (mL/mq)	35.22 ± 11.02	34.59 ± 12.35	35.55 ± 10.45	0.781
LV EF (%)	41.03 ± 11.36	37.14 ± 11.13	43.11 ± 11.11	0.090
RV EDV/BSA (mL/mq)	70.25 ± 25.66	64.59 ± 18.91	73.48 ± 28.63	0.274
RV ESV/BSA (mL/mq)	36.04 ± 20.69	35.59 ± 20.00	36.29 ± 21.42	0.915
RV SV/BSA (mL/mq)	34.21 ± 16.94	28.99 ± 24.19	37.19 ± 10.34	0.124
RV EF (%)	51.89 ± 9.69	51.61 ± 11.83	52.05 ± 8.46	0.889
nT1 PPM (ms)	1096.84 ± 104.81	1219.35 ± 102.55 *	1052.20 ± 80.45	<0.001
nT1 AL-PPM (ms)	1082.46 ± 105.65	1217.9 ± 78.6 **	1052.2 ± 80.4	<0.001
nT1 PM-PPM (ms)	1111.53 ± 103.04	1232.29 ± 123.04 ***	1080.69 ± 77.64	<0.001
nT1 RM (ms)	1.032.13 ± 37.19	1029.50 ± 29.82	1038.87 ± 39.38	0.093
nT1 IA (ms)	1264.48 ± 98.48	1233.44 ± 64.7	1281.03 ± 109.9	0.120
nT1 BP (ms)	1646.50 ± 92.75	1658.81 ± 98.7	1639.93 ± 90.5	0.517
Noise (muscle SD)	34.17 ± 7.41	34.79 ± 6.64	33.83 ± 7.87	0.680
No MVR (n)	37	13	24	0.879
MVR mild/moderate/severe (n)	8/1/0	3/0/0	5/1/0	0.892

* Only infarcted PPMs were considered; ** Only infarcted AL-PPM; *** Only infarcted PM-PPM. *p*-values are considered significant when <0.05. *iPPM: papillary muscles infarction; niPPM: papillary muscles non-infarction; MI: myocardial infarction; AL-PPM anterolateral papillary muscle; PM-PPM: posteromedial papillary muscle; LV: left ventricle; EDV/BSA: end-diastolic volume/body surface area; ESV/BSA: end-systolic volume/body surface area; SV/BSA: stroke volume/body surface area; EF: ejection fraction; RV: right ventricle; IA: infarcted area; BP: blood pool; SD: standard deviation; MVR: mitral valve regurgitation.*

**Table 3:** Length and longitudinal strain values of the PPMs.

Morpho-Dynamics Parameters (Mean ± SD)	Total (N: 46)	IPPM Group (N: 16)	niPPM Group (N: 30)	*p*-Value
Diastolic Length (mm)	42.56 ± 6.23	41.88 ± 5.92 *	42.72 ± 6.33	0.622
Systolic Length (mm)	33.69 ± 5.49	34.59 ± 6.14 *	33.48 ± 5.35	0.457
PPM-ls (%)	20.88 ± 4.94	17.56 ± 6.30 *	21.65 ± 4.26	*0.002*
AL-PPM-ls (%)	20.99 ± 4.63	16.98 ± 6.41 **	23.02 ± 4.84	*0.009*
PM-PPM-ls (%)	20.71 ± 5.32	15.18 ± 4.59 ***	21.74 ± 5.21	*0.011*

* Only infarcted PPMs were considered; ** Only infarcted AL-PPM; *** Only infarcted PM-PPM. *SD: standard deviation; iPPM: papillary muscle infarction; niPPM: papillary muscle non-infarction; mm: millimeters; PPM-ls: papillary muscle longitudinal strain; AL-PPM: anterolateral papillary muscle; PM-PPM: posteromedial papillary muscle*.

**Table 4:** The CNR values of infarcted-PPM to BP/RM and of IA to BP/RM calculated in the T1 maps.

CNR	Value
nT1 (IA/RM)	6.27 ± 2.42
nT1 (infarcted PPM/RM) *	5.87 ± 3.31
nT1 (IA/BP)	−12.56 ± 3.21
nT1 (infarcted PPM/BP) *	−12.96 ± 3.77

* Only infarcted PPMs were considered. *CNR: contrast-to-noise ratio; IA: infarcted area; RM: remote myocardium; PPM: papillary muscle; BP: intracavitary blood pool*.

## Data Availability

The dataset of the study is available from the corresponding author upon reasonable request.

## Abbreviations

AAR	Area at risk
AL-PPM	Anterolateral papillary muscles
ANOVA	Analysis of variance
AUC	Area under the curve
BMI	Body mass index
BP	Blood pool
BSA	Body surface area
CI	Confidence interval
CMR	Cardiac magnetic resonance
CNR	Contrast to noise ratio
DM	Diabetes mellitus
EDV	Indexed end-diastolic volume
EDV/BSA	End-diastolic volume/body surface area
EF	Ejection fraction
ESV	End-systolic volume
ESV/BSA	End-systolic volume/body surface area
GBCA	Gadolinium-based contrast agent
HR	Heart rate
HTN	Systemic arterial hypertension
IA	Infarcted area
ICC	Intraclass correlation coefficient
iPPM	Papillary muscle infarction
LAD	Left anterior descending artery
LCX	Left circumflex artery
LGE	Late gadolinium enhancement
LV	Left ventricle
MI	Myocardial infarction
MR	Magnetic resonance
MV	Mitral valve
MVO	Microvascular obstruction
MVR	Mitral valve regurgitation
niPPM	Non infarcted papillary muscle
OM	Obtuse marginal branch
PPM	Papillary muscle
PPM-ls	Longitudinal strain of papillary muscle
PM-PPM	Posteromedial papillary muscles
RCA	Right coronary artery
RM	Remote myocardium
ROC	Receiver operating characteristic
ROI	Region of interest
RV	Right ventricular
RVR-AF	Rapid ventricular response-atrial fibrillation
SD	Standard deviation
STEMI	ST elevation myocardial infarction
STIR	Short Tau inversion recovery
SV	Stroke volume
SV/BSA	Stroke volume/body surface area
TE	Time to echo
TR	Repetition time
TTE	Transthoracic echocardiogram
